# Single-stage extremity reconstruction through the use of dermal matrices: the power of Integra^®^ bilayer wound matrix in the face of medical comorbidities, patient preference and non-compliance

**DOI:** 10.1080/23320885.2022.2047052

**Published:** 2022-03-14

**Authors:** Ajul Shah, Philippe Taupin

**Affiliations:** a The Plastic Surgery Center, Institute for Advanced Reconstruction, Shrewsbury, NJ, USA; b Medical Affairs, Integra LifeSciences, Princeton, NJ, USA

**Keywords:** Acellular dermal matrices, patient compliance, medical comorbidities, patient populations, wound reconstruction

## Abstract

We report 4 cases of extremity reconstruction using Bilayer Wound Matrix with plan to perform two-stage procedures. Patient preference or non-compliance led to single-stage reconstruction with wound re-epithelialization. In this setting, dermal matrices may be used as single-stage stand alone wound reconstruction procedures, even in patients with comorbid conditions. Ajul Shah is a consultant of Integra LifeSciences Corporation Philippe Taupin is an employee of Integra LifeSciences Corporation.

## Introduction

Extremity reconstruction remains a challenge for plastic surgeons because of optimal aesthetic and functional outcomes. When facing wound reconstruction, trauma and plastic surgeons have different options to choose from, including skin grafting, local, regional, distant pedicled and free flaps, and tissue expansion [1]. The choice of which will depend on the anatomical characteristics of the lesion, its size, depth, location, local and surrounding tissue quality, patient comorbidities and preferences [2]. Skin grafting is limited by its capacity to cover bone devoid of periosteum, tendon devoid of paratenon, exposed hardware, and/or contaminated wounds. Advanced technologies, such as dermal matrices, are now a new rung on and integral part of the reconstructive ladder, adding mobility and durability to skin grafting [3,4].

Integra^®^ Dermal Regeneration Template (IDRT; Integra LifeSciences, Princeton NJ, USA) and its variant, Integra^®^ Bilayer Wound Matrix (IBWM), are acellular dermal matrices (ADMs) [5]. ADMs are a class of biological, synthetic, and composite scaffold materials used to augment and replace deficient or missing skin and soft tissues [6]. IDRT and IBWM are xenogenic bioengineered collagen matrices composed of a porous layer of cross-linked type I collagen-glycosaminoglycans, covered with a semi-porous layer of silicone [7]. The bilayer matrices are generally applied to the wound in a two-stage procedure [8]. First, the wound is excised and debrided, and the dermal matrix is placed over the wound bed. Second, after 3–4 weeks, when neovascularization is achieved and the neodermis is being formed, the silicone layer is removed and replaced by a split-thickness thin graft (STSG) [9]. The collagen matrix provides structure and stability, and enables infiltration of macrophages, fibroblasts, lymphocytes and endothelial cells, resulting in the generation of a new vascular network and the formation of a neodermis. The temporary silicone layer serves to control moisture loss from the wound and minimize bacterial invasion. Two-stage reconstruction requires patients to undergo two surgical procedures and necessitates patient compliance. ADMs, such as IDRT and IBWM, offer a novel approach for wound reconstruction, with less donor sites morbidity, and advantages over local/regional and free tissue transfer [6]. Local and regional flaps are limited by their capacity to cover large defects. Local flaps, regional flaps, pedicled and free flaps are associated with substantial donor-site morbidity, and prolonged surgery time and hospital stay [1]. Over the years, IDRT and IBWM, and their meshed variants, have been used to treat a broad range of wounds from burns, acute and chronic wounds, cancer resection, scar reconstruction to extremity reconstruction on patients with large defects [10–18].

In this article, we report a series of 4 adult patients managed for extremity reconstruction using Integra^®^ Meshed Bilayer Wound Matrix (IMBWM), with the aim to perform two-stage procedures. Either patient preference or non-compliance led to re-epithelialization of the wounds in a single-stage procedure, thereby obviating the need for two-stage reconstruction.

## Case reports

The study was conducted following the principles outlined in the Declaration of Helsinki. All patients were informed of the pros and cons of IMBWM treatment, and signed a consent approving or rejecting such information. All patients gave informed written consent for the use of the data collected.

### Case 1

**Figure 1.**
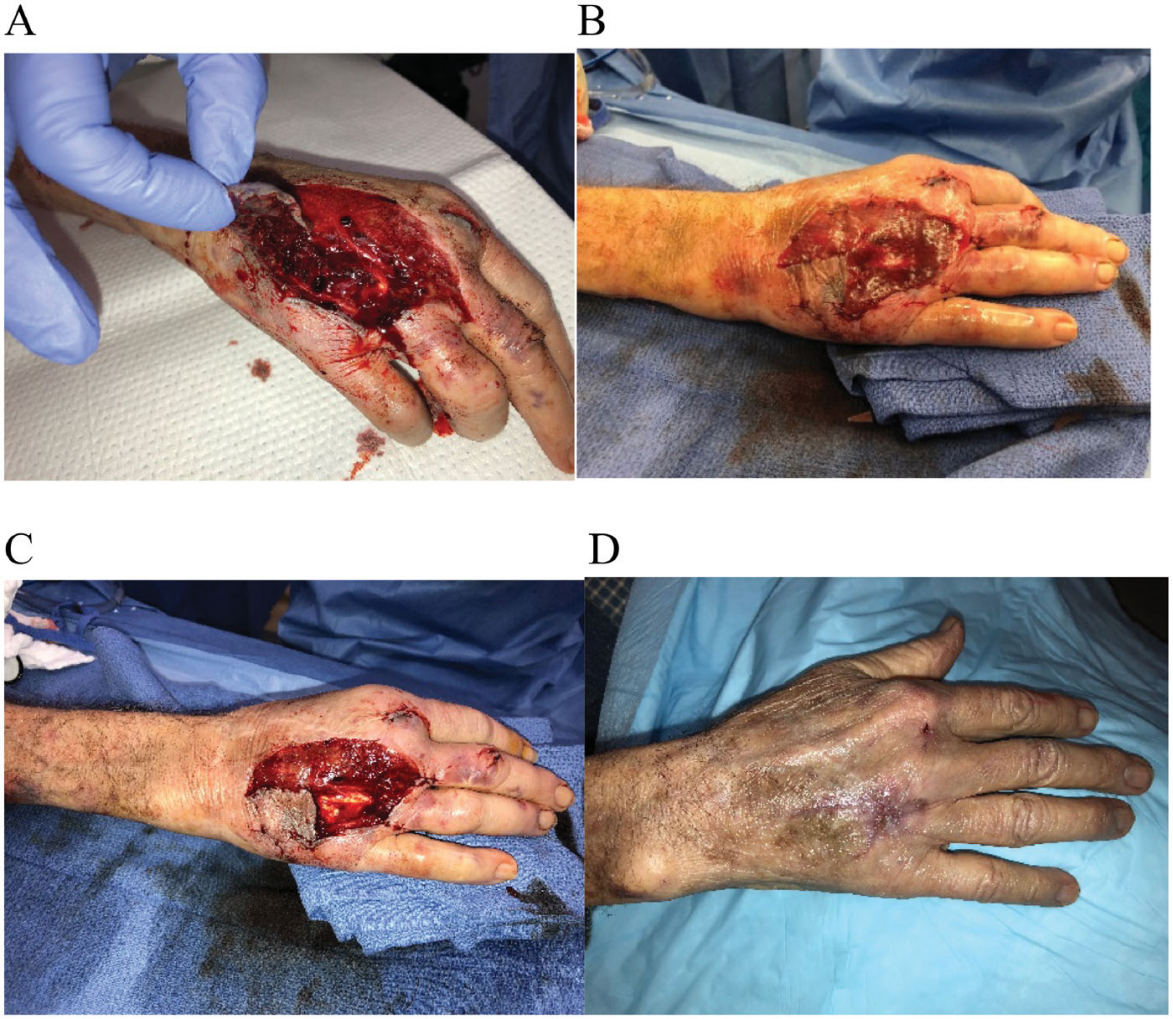
Case 1. An 80-year-old female had a motor vehicle collision. She suffered an open fracture of the 4^th^ metacarpal with soft tissue loss on the dorsum of the right hand and exposed extensor tendons (A). The patient underwent 1 operative debridement and a sheet of IMBWM was applied to the wound (B). Vascularization of the dermal matrix was achieved 3 weeks after the first surgical procedure, at which time the silicone layer was removed (C). Matrix take was 100%. Due to patient preference, including a desire to avoid repeat surgery and anesthesia, the wound was subsequently treated to allow healing by secondary intention. Six weeks after matrix placement, complete coverage of the wound by re-epithelialization was observed (D).

An 80-year-old female, with a significant cardiac history, had a motor vehicle collision. She was admitted to the hospital and presented with an open fracture of the 4^th^ metacarpal of the right hand with soft tissue loss on the dorsum and exposed extensor tendons (Figure 1(A)**)**. The patient was placed on intravenous (IV) antibiotics at the time of arrival and diagnosis of the injury. The wound was open and underwent a single operative debridement prior to definitive reconstruction; the resulting defect measured 5.5 × 4 cm. During the same operation with the debridement, IMBWM was applied to the wound under general anesthesia, and the sheet of dermal matrix was fixed in place using a running absorbable suture (Figure 1(B)). The patient was discharged the following day after placement of the matrix. Postoperative dressings included bacitracin ointment to the matrix with a non-adhering dressing and gauze wraps. Vascularization of the dermal matrix was achieved 3 weeks after placement, at which time the silicone layer was removed in the office setting (Figure 1(C)). Matrix take was 100%. IBWM was applied with the aim to perform a two-stage procedure. However, due to patient preference, including a desire to avoid repeat surgery and anesthesia, the wound was subsequently managed to allow healing by secondary intention. After silicone removal, the dressings continued in the same fashion on a daily basis. Time to healing occurred 6 weeks after matrix placement (Figure 1(D)). There was no postoperative complications. Patient was compliant with medical advice, and the final outcome revealed a well-healed wound and satisfactory cosmesis, with patient demonstrating full range of motion (ROM) and function of the hand and fingers.

### Case 2

**Figure 2.**
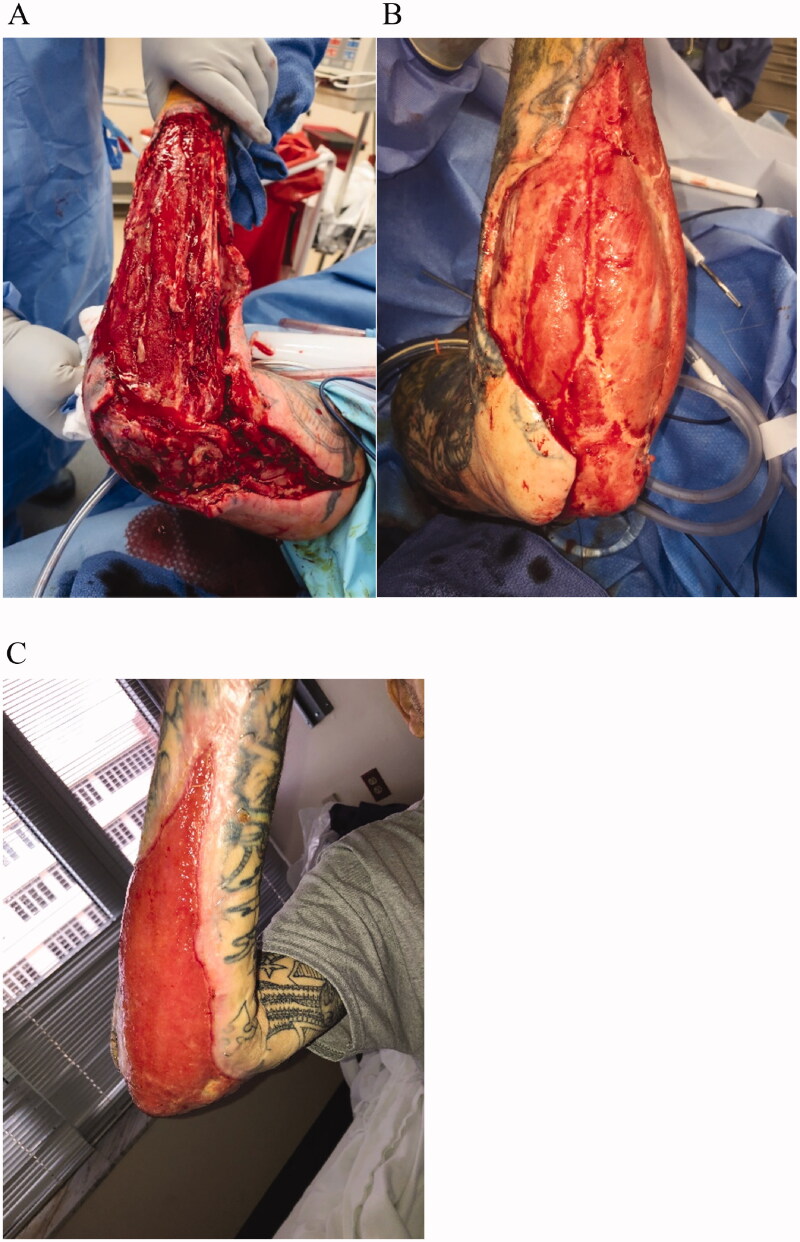
Case 2. A 53-year-old male was admitted to the hospital for sepsis and infection in the right upper extremity. Due to the sepsis condition, there was a concern the right upper extremity infection would progress into necrotizing fasciitis. The patient underwent 7 operative debridement procedures (A) and a sheet of IMBWM was applied to the wound (B). Patient was not compliant and against medical advice; he left the hospital 1 day after matrix placement and did not return to the hospital for follow up visits. Three months later, patient re-appeared to the hospital for concern of an infection in his contra-lateral hand. By that time, the silicone layer had been removed, the IMBWM had fully vascularized and fully integrated. There was significant re-epithelialization with new skin formation (C). The patient demonstrated an excellent surgical outcome with full ROM of the elbow (C).

A 53-year-old male was admitted to the hospital for sepsis and infection in the right upper extremity. Patient had a history of IV drug use, previous endocarditis, and multiple incisions and drainages for a variety of infections. There was an immediate concern for necrotizing fasciitis that was confirmed. Subsequently, the wound underwent 7 operative debridement procedures prior to eventual control of the infection over the course of 14 days. The patient was placed on culture directed antibiotic therapy and returned to the operating room (OR) every 48 h. Following debridement, significant wound ensued inclusive of upper arm, elbow, forearm, and wrist, with exposed muscle throughout; the resulting defect measured 23 × 45 cm (Figure 2(A)). IMBWM was applied to the wound in the OR under general anesthesia after the wound was deemed free of infection (Figure 2(B)). The sheet of dermal matrix was fixed in place using multiple staples, and the postoperative dressing included bacitracin ointment to the matrix with a non-adhering dressing and gauze wraps. The planned postoperative dressings were to be the same performed on a daily basis. Unfortunately, the patient was non-compliant with medical advice and left the hospital 1 day after matrix placement; he did not return to the hospital for follow up visits. Three months later, patient re-appeared to the hospital for concern of an infection in his contra-lateral hand. By that time, the silicone layer had been removed. The patient stated he was intermittently placing a moisturizing ointment to the surgical reconstruction. Despite patient non-compliance, the IMBWM had fully vascularized and fully integrated. Furthermore, there was significant re-epithelialization with new skin formation (Figure 2(C)). There was no infectious and no postoperative complications noted. The patient demonstrated an excellent surgical outcome despite extreme non-compliance, with full ROM of the elbow (Figure 2(C)). Of note, the patient was scheduled for secondary skin grafting during his repeat hospitalization, and unfortunately left against medical advice the morning he was supposed to have final reconstruction performed. He was not heard from again.

### Case 3

**Figure 3.**
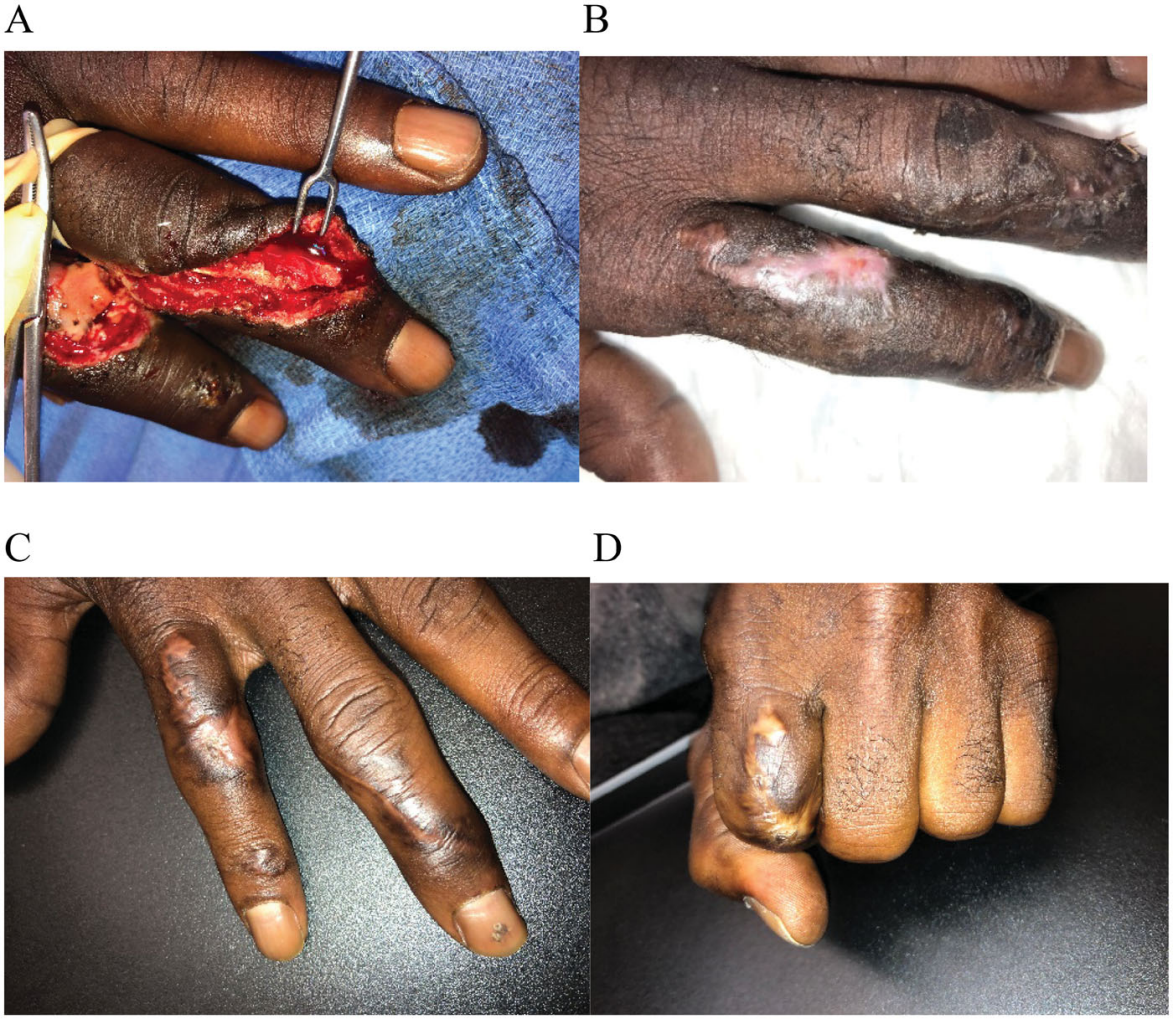
Case 3. A 65-year-old male admitted to the hospital after a table saw injury. He suffered injuries to his dorsal 2^nd^ and 3^rd^ fingers with the left 2nd finger down to bone and no periosteum (A). The dorsal 2^nd^ finger was debrided and a sheet of IMBWM applied to the wound. Vascularization of the dermal matrix on the dorsal 2^nd^ finger was achieved 3 weeks after placement (B), at which time the silicone layer was removed. Due to patient preference and the desire to allow for time for sequential vascularization of the IMBWM to occur, the wound was subsequently allowed to heal by secondary intention. Patient recovered full ROM on his 2^nd^ finger after 12 weeks of treatment with a fully healed reconstruction (C and D).

A 65-year-old male injured his left arm with a table saw. He was admitted to the hospital with injuries to his dorsal 2^nd^ and 3^rd^ fingers with the injury to the left 2^nd^ finger inclusive of a unicortical fracture of the proximal phalanx with an absence of periosteum, as well as non-viable skin and subcutaneous tissue (Figure 3(A)). Patient had a history of uncontrolled hypertension, diabetes, and was a heavy smoker. The patient was placed on administration of IV antibiotics. The injury on the dorsal 3^rd^ finger included a zone 1 extensor tendon injury with laceration and was repaired at the time of surgical intervention. The wound on the dorsal 2^nd^ finger underwent 1 operative debridement procedure under general anesthesia, inclusive of debridement of non-viable skin and subcutaneous tissue which left a defect measuring 3 × 2 cm inclusive of exposed bone. IMBWM was applied to the wound on the dorsal 2^nd^ finger after debridement. The sheet of dermal matrix was fixed in place using a running absorbable suture. Postoperative dressing was bacitracin ointment, a non-adhering dressing, a small gauze wrap, and a volar splint to protect the reconstruction of the 3^rd^ finger. The patient was discharged the same day after placement of the matrix. The postoperative dressing was left in place as part of the splint and overall treatment plan. Vascularization of the dermal matrix was achieved 3 weeks after placement (Figure 3(B)), at which time the silicone layer was removed in the office without anesthesia. Matrix take was approximately 60% at that time. IMBWM was applied with the aim to perform two-stage procedures. However, due to patient preference and the desire to allow for time for sequential vascularization of the IMBWM to occur, the wound was subsequently allowed to heal by secondary intention [19]. Daily dressing changes with bacitracin ointment, a non-adhering dressing, and a small gauze wrap were instituted in addition to start of occupational hand therapy. There was no postoperative complications. Patient was compliant with medical advice. Patient recovered full ROM on his 2^nd^ finger after 12 weeks of treatment with a fully healed reconstruction and satisfactory cosmesis (Figure 3(C and D)).

### Case 4

**Figure 4.**
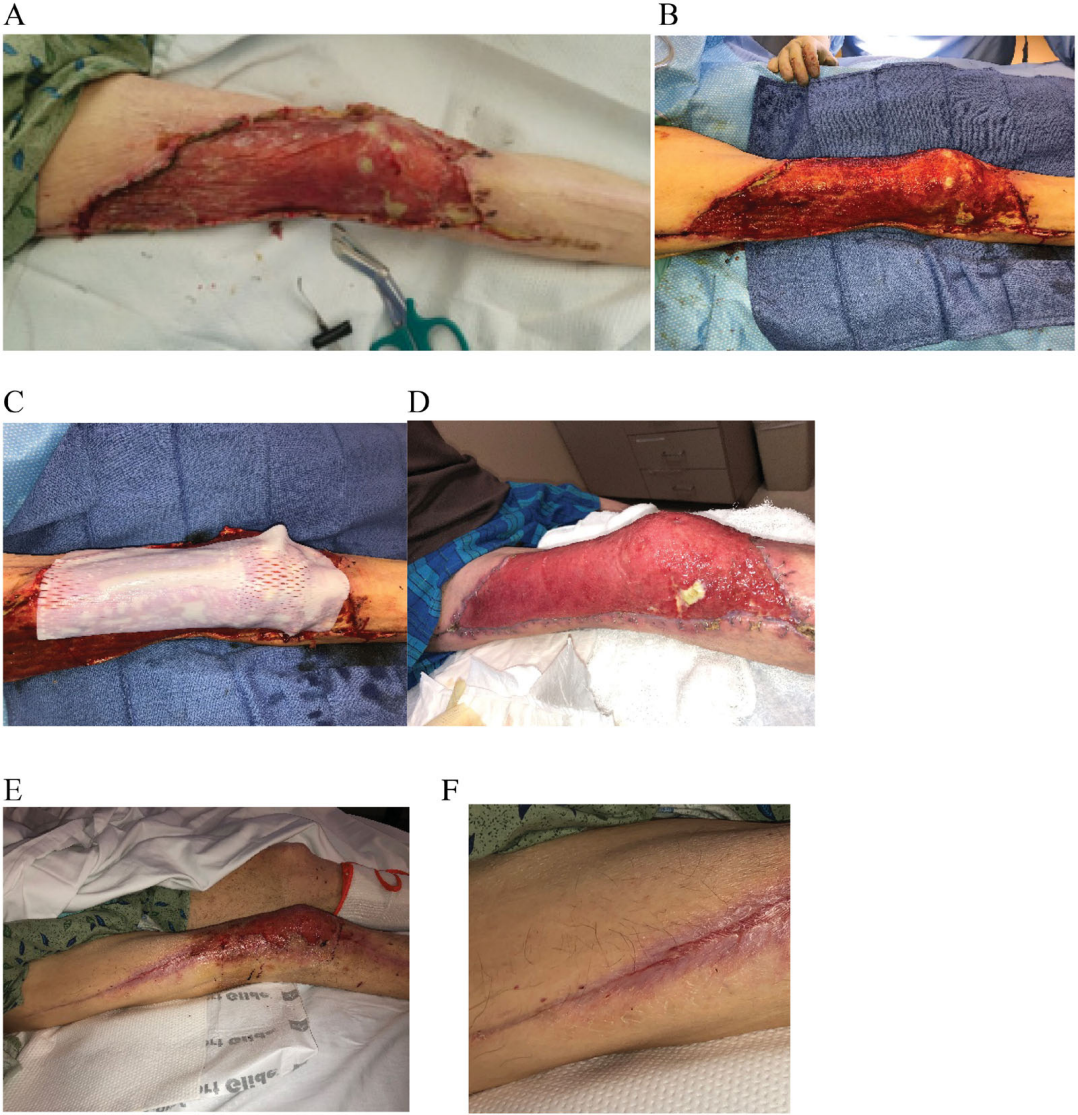
Case 4. A 62-year-old male admitted to the hospital for DKA and extensive necrotizing fasciitis on the right leg with extensive tissue loss. He presented an extensive lower extremity wound of the right thigh from below the knee to the hip joint and extending medially toward the right groin. IMBWM was applied to the debrided wound in combination with VAC therapy (A). Due to the desire to augment soft tissue over the knee, as well provide additional soft tissue bulk, a sheet of meshed PriMatrix was stacked over the neodermis (B and C). Two weeks after VAC placement, full integration and vascularization of the PriMatrix was noted (D). The patient was scheduled for secondary skin grafting to complete the reconstruction. However, the patient was not compliant with follow up and did not return for multiple months. Six months later, patient was readmitted to the ICU, upon wound evaluation, over 70% of the wound had fully healed and re-epithelialized (E and F). Dermal appendages such as hair follicles had returned (F).

A 62-year-old male was admitted to the hospital for diabetic ketoacidosis (DKA) and extensive necrotizing fasciitis on the right leg with extensive tissue loss. The patient was initially admitted to the intensive care unit (ICU) for wound management, sepsis and pain control. After debridement by orthopedic surgery and trauma surgery, the patient presented with an extensive lower extremity wound on the right thigh from below the knee to the hip joint and extending medially toward the right groin. The resulting defect measured approximately 30 × 55 cm. IMBWM was applied to the wound, by orthopedic surgery, in combination with vacuum-assisted closure (VAC) therapy (Figure 4(A)). Due to the extensive nature of the wound in the face of poorly controlled diabetes, the patient was transferred to our care for continued reconstruction (Figure 4(B)). Due to the desire to augment soft tissue over the knee, as well provide additional soft tissue bulk, a sheet of meshed PriMatrix (meshed 2:1) was serially applied over the neodermis, placed in the OR under general anesthesia (Figure 4(C)). PriMatrix^®^ (Integra LifeSciences, Princeton NJ, USA) is a single-layer ADM-derived from fetal bovine dermis. The sheet of PriMatrix was fixed in place using staples. The initial postoperative dressing consisted of a wound VAC which was affixed to the wound after a contact barrier of a non-adhering dressing was placed, and the wound VAC was set to − 125 mm Hg suction (KCI, St. Paul MN, USA). The patient was discharged, was performing every other day VAC dressing changes, and was followed up 2 weeks after VAC placement. The VAC was removed, and full integration and vascularization of the PriMatrix were noted (Figure 4(D)). At that time, the patient was scheduled for secondary skin grafting to complete the reconstruction, and daily dressing changes with bacitracin ointment, a non-adhering dressing, and gauze wraps were instituted. However, the patient was non-compliant with follow up and did not return for multiple months. Six months later, patient was readmitted to the ICU for altered mental status and DKA. Initial blood glucose level on arrival was read at 750, indicating the high likelihood that over the past 6 months, glucose levels were likely highly uncontrolled. Nonetheless, upon wound evaluation, over 70% of the wound had fully healed and re-epithelialized, despite the extreme non-compliance and severe diabetes (Figure 4(E and F)). Furthermore, dermal appendages such as hair follicles had returned (Figures 4(F)). The patient was again scheduled for skin grafting after control of his DKA, and unfortunately again left against medical advice. He was not heard from again.

## Discussion

We report a case series of 4 adult patients managed for soft tissue defects of the extremity using IMBWM. While the 4 patients were scheduled for two-stage procedures, patient preference or non-compliance led to the re-epithelialization of the wounds by secondary intention.

The 4 patients (age range 53–80-year-old) reported had comorbidities, i.e. cardiac history, IV drug use and previous endocarditis, uncontrolled hypertension, diabetes and heavy smoker, and DKA and extensive necrotizing fasciitis, which did not affect the successful integration of the dermal matrix. In the 4 patients managed using IMBWM, the dermal matrices integrated successfully in the various extremity wound types treated, i.e. open fracture of the hand with soft tissue loss and exposed extensor tendons, infection in the upper extremity, injuries to fingers and extensive necrotizing fasciitis on the leg. In all, for these 4 patients, the integration of the matrices in the wound beds was not affected by the patients’ profiles, i.e. comorbidities, wound etiology, previous infection, anatomy and wound characteristics.

IDRT and IBWM are bilayer dermal matrices bioengineered from adult bovine Achilles tendons and chondroitin-6-sulfate derived from shark cartilage, and have different indications for use in the United States. Vascularization of the neodermis after Integra bilayer dermal matrix placement is achieved when the color of the dermal matrix has progressed from pink through pale yellow and finally to peach [9]. We used this color change to assess vascularization of IMBWM in the 4 cases reported. In patients 1 and 3, the matrices revascularized successfully and the silicone layers detached within 2–3 weeks after matrix placement, in accordance with previous reports [9]. While in patient 1, 100% matrix take was observed 2–3 week after matrix placement, in patient 3 matrix take was approximately 60% at that time. In this latter patient, the wound was subsequently allowed to heal by secondary intention allowing for time for sequential vascularization of the IMBWM to occur [19]. Due to non-compliance, these data in not available for patient 2 who left the hospital 1 day after matrix placement, and for patient 4 who was managed initially by a different service. Management of the wounds of patients 1 and 3 using IMBWM led to complete healing by re-epithelialization, with satisfactory cosmesis and full ROM recovery after surgery. Management of the wounds of patients 2 and 4 using IMBWM led to healing by re-epithelialization. However, due to patients’ non-compliance and lost to follow up, these 2 patients were not followed to complete healing.

In the 4 cases reported, the use IMBWM led to the successful re-epithelialization and reconstruction of the skin with patients recovering ROMs. IDRT and IBWM allow extra thickness of soft tissue reconstruction for exposure areas of great tension and pressure, such as the elbow and knee, and provide more durability than just skin graft alone (patients 2 and 4). IDRT and IBWM provide high quality coverage over wounds of various etiologies with exposed bone or tendon, where it is efficient in supporting the formation of a new vascularized dermis, and provides a higher likelihood of take by sequential vascularization considering the hostile environment it was placed in (patients 1 and 3) [19–21]. The healed wound with exposed structures were reported as very pliable and uniform in color and texture, and not adhering to the deeper structures, providing a gliding plane for tendons particularly [12]. Stacking dermal matrices is a mean to obtain enough volume for reconstruction [22]. The stacking of IBWMs serially has been reported to address defect over the palm of the hand in terms of depth, leading to excellent wound healing and coverage and full ROM [13]. In Patient 4, due to the desire to augment soft tissue over the knee, as well provide additional soft tissue bulk, a sheet of meshed PriMatrix was applied serially over the neodermis after IMBWM placement. PriMatrix is a decellularized dermal matrix-composed primarily of type I and type III collagen; type III collagen is associated with healing and developing tissues [23]. Two weeks after VAC placement, full integration and vascularization of the PriMatrix were noted. Our results show that IMBWM, as stand alone or with stacking dermal matrix serially (with meshed PriMatrix), without subsequent skin grafting led to the reconstruction of wounds not adhering to the deeper structures, providing a gliding plane for tendons particularly.

Due to patient preference or non-compliance, the 4 patients we reported underwent wound closure using IMBWM by single-stage and re-epithelialization, and not by the scheduled for two-stage procedure. Previous studies have utilized a single-stage reconstructive approach using IBWM without subsequent skin grafting, including for the reconstruction of defects ≥100 cm^2^ [24–27]. Seth et al. reported single-stage reconstruction for nasal reconstruction using IBWM without applying a skin graft. The most common reason for single-stage reconstruction with re-epithelialization was patient preference to avoid an autologous reconstruction, followed by large defect size/depth. Average time for complete re-epithelialization was 1.5 ± 0.8 months [28]. For patients for whom the single-stage procedure was conducted due to patient preference (patients 1 and 3), these patients were monitored closely during the re-epithelialization process, and followed medical advices and indications for wound management. In those patients, we reported time for complete re-epithelialization of 6 weeks for a defect measured 5.5 × 4 cm (patient 1), and 12 weeks for a defect measured 3 × 2 cm inclusive of exposed bone (patient 3). In patient 3, the extended time to healing of 12 weeks may be attributed for sequential vascularization of the IMBWM to occur [19]. For patients for whom the single-stage procedure occurred due to patient non-compliance (patients 2 and 4), we reported significant re-epithelialization with new skin formation, despite the extreme non-compliance. All 4 patients did not develop contractures, as previously reported for single-stage re-epithelialization using IBWM [28]. In addition, we reported in one patient (patient 4), dermal appendages such as hair follicles had returned. In all, our data revealed single-stage healing with re-epithelialization, with regeneration rather than scaring.

Single-stage reconstruction with IBWM and re-epithelialization, without subsequent skin grafting, when conducted as per patient preference, led to successful re-epithelialization and reconstruction of the skin with patients recovering full ROMs, when the patients followed postoperative medical advices and indications. These postoperative medical advices and indications, e.g. daily dressing changes, were well tolerated by the patients. With regards to single-stage reconstruction with IBWM and re-epithelialization, without subsequent skin grafting, when occurring as a result of non-compliance, our results showed significant re-epithelialization with new skin formation in patients whom with other reconstructive procedures may have led to otherwise unsuccessful reconstructions. Our results highlight the benefits of dermal matrices for adding mobility and durability to skin grafting in the reconstruction ladder/elevator [4]. Results from these 4 cases highlight the power and resilience of IBWM in the face of medical comorbidities, patient preference and non-compliance.

The use of ADMs in extremity reconstruction often involves two-stage reconstruction. Two-stage procedures are generally conducted on patients with large defects who would benefit from a secondary skin graft to complete wound closure. On the one hand, there are decades of literature evidence showing the benefits of two-stage procedures for restoring form and function to patients. On the other hand, two-stage procedures require the patients to undergo two surgical procedures and necessitate patient compliance. Single-stage procedures, through re-epithelialization, offer the opportunity to avoid multiple operations and reduce donor-site morbidity. Our results show that, if possible, single-stage procedures through re-epithelialization may be preferable for patients, based on patient preference or non-compliance, including for large wounds.

## Conclusion

ADMs are integral part of the reconstructive armamentarium. Results from this 4-case series highlight the power and resilience of dermal matrices and single-stage reconstruction with wound re-epithelialization in the face of medical comorbidities, patient preference and non-compliance. Single-stage procedures remove the reliance on patient compliance in the reconstructive algorithm, a welcome finding in difficult patient populations.
